# Global Evolution of Research in Artificial Intelligence in Health and Medicine: A Bibliometric Study

**DOI:** 10.3390/jcm8030360

**Published:** 2019-03-14

**Authors:** Bach Xuan Tran, Giang Thu Vu, Giang Hai Ha, Quan-Hoang Vuong, Manh-Tung Ho, Thu-Trang Vuong, Viet-Phuong La, Manh-Toan Ho, Kien-Cuong P. Nghiem, Huong Lan Thi Nguyen, Carl A. Latkin, Wilson W. S. Tam, Ngai-Man Cheung, Hong-Kong T. Nguyen, Cyrus S. H. Ho, Roger C. M. Ho

**Affiliations:** 1 Institute for Preventive Medicine and Public Health, Hanoi Medical University, Hanoi 100000, Vietnam; 2 Bloomberg School of Public Health, Johns Hopkins University, Baltimore, MD 21205, USA; carl.latkin@jhu.edu; 3 Center of Excellence in Artificial Intelligence in Medicine, Nguyen Tat Thanh University, Ho Chi Minh City 700000, Vietnam; giang.coentt@gmail.com (G.T.V.); ngaiman_cheung@sutd.edu.sg (N.-M.C.); 4 Center of Excellence in Evidence-based Medicine, Nguyen Tat Thanh University, Ho Chi Minh City 700000, Vietnam; nurtwsw@nus.edu.sg; 5 Institute for Global Health Innovations, Duy Tan University, Da Nang 550000, Vietnam; giang.ighi@gmail.com (G.H.H.); huong.ighi@gmail.com (H.L.T.N.); 6 Center for Interdisciplinary Social Research, Phenikaa University, Yen Nghia, Ha Dong District, Hanoi 100803, Vietnam; hoang.vuongquan@phenikaa-uni.edu.vn (Q.-H.V.); tung.homanh@phenikaa-uni.edu.vn (M.-T.H.); lvphuong@gmail.com (V.-P.L.); toan.homanh@phenikaa-uni.edu.vn (M.-T.H.); 7 Faculty of Economics and Finance, Phenikaa University, Yen Nghia, Ha Dong district, Hanoi 100803, Vietnam; 8 Sciences Po Paris, Campus de Dijon, 21000 Dijon, France; thutrang.vuong@sciencespo.fr; 9 Vietnam-Germany Hospital, 16 Phu Doan street, Hoan Kiem district, Hanoi 100000, Vietnam; kimcuongvd@gmail.com; 10 Alice Lee Centre for Nursing Studies, Yong Loo Lin School of Medicine, National University of Singapore, Singapore 119228, Singapore; 11 Information Systems Technology and Design (ISTD) pillar, Singapore University of Technology and Design, Singapore 487372, Singapore; 12 A.I. for Social Data Lab (AISDL), Vuong & Associates, 3/161 Thinh Quang, Dong Da District, Hanoi 100000, Vietnam; htn2107@caa.columbia.edu; 13 Department of Psychological Medicine, National University Hospital, Singapore 119228, Singapore; cyrushosh@gmail.com; 14 Center of Excellence in Behavioral Medicine, Nguyen Tat Thanh University, Ho Chi Minh City 700000, Vietnam; pcmrhcm@nus.edu.sg; 15 Department of Psychological Medicine, Yong Loo Lin School of Medicine, National University of Singapore, Singapore 119228, Singapore; 16 Biomedical Global Institute of Healthcare Research & Technology (BIGHEART), National University of Singapore, Singapore 117599, Singapore

**Keywords:** bibliometric analysis, artificial intelligence, health, medicine, global, mapping, AI ethics

## Abstract

The increasing application of Artificial Intelligence (AI) in health and medicine has attracted a great deal of research interest in recent decades. This study aims to provide a global and historical picture of research concerning AI in health and medicine. A total of 27,451 papers that were published between 1977 and 2018 (84.6% were dated 2008–2018) were retrieved from the Web of Science platform. The descriptive analysis examined the publication volume, and authors and countries collaboration. A global network of authors’ keywords and content analysis of related scientific literature highlighted major techniques, including Robotic, Machine learning, Artificial neural network, Artificial intelligence, Natural language process, and their most frequent applications in Clinical Prediction and Treatment. The number of cancer-related publications was the highest, followed by Heart Diseases and Stroke, Vision impairment, Alzheimer’s, and Depression. Moreover, the shortage in the research of AI application to some high burden diseases suggests future directions in AI research. This study offers a first and comprehensive picture of the global efforts directed towards this increasingly important and prolific field of research and suggests the development of global and national protocols and regulations on the justification and adaptation of medical AI products.

## 1. Introduction

While the growing importance and relevance of artificial intelligence (AI) is indisputable, the term itself has no universally agreed upon definition [1,2]. AI commonly refers to the computational technologies that mimic or simulate processes supported with human intelligence, for instance, reasoning, deep learning, adaptation, interaction, and sensory understanding [1]. In a broader definition, the Cambridge dictionary puts AI as an interdisciplinary approach that adopts principles and devices from a variety of fields, such as computation, mathematics, logics, and biology, to solve the problem of understanding, modeling, and replicating intelligence and cognitive processes [3]. As such, applications of AI can be found in various domains, from robotics [4,5], image and voice recognition [6], to natural language processing and expert systems [2]. Given its broad, dynamic and rapidly growing capabilities, it is no wonder that AI has been applied in the field of medicine since as early as the 1950s when physicians made the first attempts to improve their diagnoses using computer-aided programs [3]. A notable example of this is abdominal pain diagnosis that utilized computer analysis by Gunn in 1976 [7,8]. The interest and advances in medical AI applications have surged in recent years, thanks to the substantially enhanced computing power of modern computers [4,9] and the vast amount of digital data now available for collection and utilization [1].

Within the medical literature, scholars have written extensively on the benefits of AI applications, highlighting the technology’s potential to improve diagnostic and therapeutic accuracy and the overall clinical treatment process [10,11]. With its sophisticated algorithms and deep learning capacity, AI applications have assisted doctors and medical professionals in general in the domains of health information systems, geocoding health data, epidemic and syndromic surveillance, predictive modeling and decision support, and medical imaging [4,5,12,13]. In particular instances, an AI system can provide health professionals with constant, possibly real-time updates of medical information from various sources including journals, textbooks, clinical practices, and patients to inform proper patient care [14] and enable appropriate inferences for health risk alert and health outcome prediction [15].

As AI is rapidly transforming the medical landscape, scholarship on the topic has also mounted substantially in recent years, presenting the need for a comprehensive review of the research patterns as well as trends of AI in medicine (AIM). In their thorough review article on Nature Biomedical Engineering, Yu, Beam, and Kohane [4] survey the literature on AIM, explain the advanced techniques and their applications, and point out the breakthroughs and challenges for the field. The paper, though among the most recent attempts to draw out clinical integration of medical AI at various stages, has yet to dig into the entirety of the literature on AIM over a certain period of time. Thus, in order to identify research gaps and facilitate the clear, on-point translation of knowledge that would better inform policy development, this study presents the use of scientometric analysis in exploring research trends in the subject of AI in health and medicine. Scientometrics uses databases of published literature to objectively assess the impact of research knowledge on health issues and provide substantial empirical evidence. It shows the way of changing concerned research topics in national and international contexts with the increasing number of published articles over time, and reflects the visual collaborations of researcher networks within different topics [16,17,18]. Scientometric methods are particularly useful in the evaluation of global scientific production and development trends, such as in the cases of health systems research [19], administrative healthcare database [20], or diabetes research in Middle East countries from 1992 to 2012 [21], to name a few. Through an extensive review of the scholarship on AIM, this paper aims to present a better understanding of publications and research trends, and suggests potential directions toward solving this ongoing challenge. Specifically, we reviewed the global growth of research production in medical AI and analyzed patterns of research areas and trends in this field.

## 2. Materials and Methods

The search used the Web of Science (WOS) database from Clarivate [22] and Scopus from Elsevier [23]. WOS was chosen because it covers (i) more research fields compared with PubMed, and (ii) research dated from 1900 to the present. For the Scopus database, due to the restriction for not downloading completed data larger than 2000 papers, we could only download papers by year. This analysis focuses on articles published from 1971 to 31 December 2018 in peer-reviewed journals. The bibliometric study does not include grey literature, conference proceedings, or books/book chapters. Articles written in any languages other than English are excluded.

### 2.1. Search Strategy

There are two steps conducted in sequential order: inclusionary step, followed by an exclusionary step. Each step is explained in detail below. We applied two steps for both WOS and Scopus.

#### 2.1.1. Inclusion Step

The literature from the WOS database was retrieved using a developed set of search terms, focusing on (1) AI types, and (2) health and medicine. The search terms were chosen based on our research on prevailing literature on the topic, discussions within our team, and suggestions provided by AI experts. The team defined clearly the synonyms for search terms and resolved any potential differences via discussion. The search query is outlined in Box 1.

Box 1 **Search query text.**

(1) “Artificial intelligence” OR “Machine intelligence” OR “artificial neutral network*” OR “Machine learning” OR “Deep learn*” OR “Natural language process*” OR “Robotic*” OR “thinking computer system” OR “fuzzy expert system*” OR “evolutionary computation” OR “hybrid intelligent system*”
(2) disease* OR illness OR health-related OR medic* OR “medical diagnosis” OR treatment OR health* OR wellness OR well-being

In our final step, we connected query 1 to 2 with the “AND” operator (see Tables S1 and S2 Supplementary).

#### 2.1.2. Exclusion Step

The team excluded articles published from 1 January 2019 onwards as any capture from that period forward would include incomplete bibliometric data for that year. Other types of documents being excluded are book chapters and conference proceedings, plus items with anonymous authors and studies written in any languages other than English.

### 2.2. Data Extraction

As a restriction from Scopus, we applied this step and the following only for the dataset downloaded from WOS. Retrieved data were exported from the WOS database under text format and applied STATA version 15 (STATA Corp., College Station, TX, USA) to merge data files and extract others. Based on the Global burden of disease 2017 study, we identified 25 diseases with the highest Disability-Adjusted Life Years (DALYs) and used STATA to extract the number of papers related to AI types. The .dta file then was stored in Excel. The data exported include: (1) Title name, (2) Names of journals, (3) Authors’ name with the Web of Science affiliation, (4) Number of citations, (5) The types of documents, (6) The year of publication for each publication, (8) Author and Web of Science keywords, and (9) Abstracts.

### 2.3. Data Analysis

We applied STATA to perform a regression model for the growth of world publication in AI in healthcare and medicine.

In terms of coauthorship analysis, the study examined the most productive countries based on the number of papers, total citations, citations per paper, the number of downloaded papers, collaborative country, and international collaborative papers.

VOSviewer software was used to create visualization maps (http://www.vosviewer.com/). For the most prolific countries, we applied the cutoff point of 5 papers, and there were 93 countries in the mapping analysis.

A network graph illustrates the connection among the 568 most common authors’ keywords by applying the specific threshold of 15 appearances for each keyword. Based on this graph, the team identified the main topic of AIM.

After that, STATA was applied to the number of papers related to the AI tools and the clinical application of AI. This measure shows not only the trend applying a specific AI type for a disease, but also identifies whether the investment and testing of an AI tool for a particular disease are adequate with the burden of disease (in this study we used disability-adjusted life-years or DALYs).

## 3. Results

### 3.1. The Publication Trend

**Figure 1:**
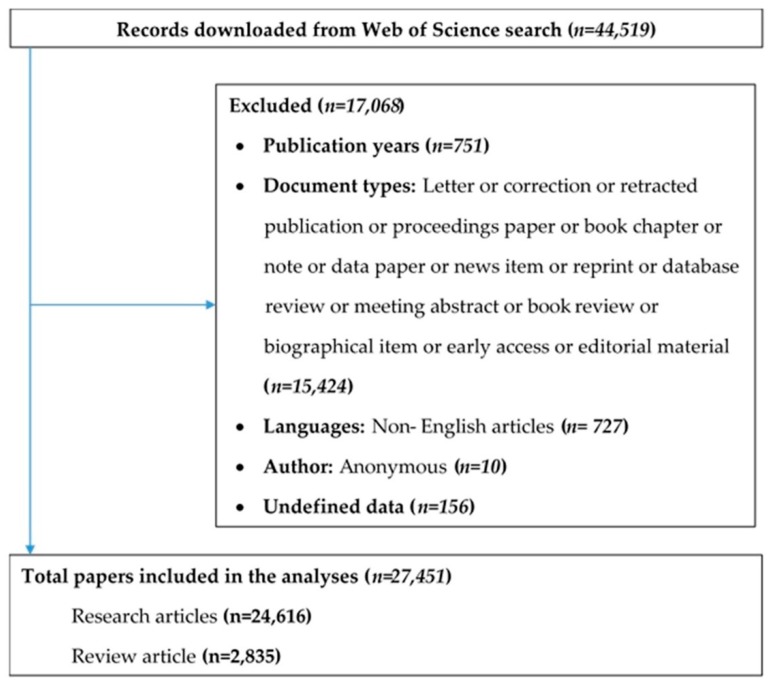
Selection of papers in the Web of Science database.

After the removal of unmatched data (15,197 research results), 27,451 research results (24,758 Article and 2849 reviews) were included from WOS published between 1971 and 2018. (Figure 1)

Table 1 shows the distinctive transformation of worldwide publications on AI in medicine and health. Below are some of the highlights:

- Most of the papers (80.0%) were assorted into one (*n* = 14,756; 53.6%) or two (*n* = 7225; 26.4%) subject categories.
- The number of publications dated between 2008 and 2017 (16,913 articles) accounts for 61.6% of the total number of publications being analyzed. This figure was double compared with the previous time range and seven times as much as that in the previous ten-year period.
- The number of countries means the paper was written by one country only or in collaboration with others. Based on that information, we found that AI-related medical research was mainly performed by one to three countries (85.9%). The global collaboration among nation-states was not so high (14.0%).

**Figure 2:**
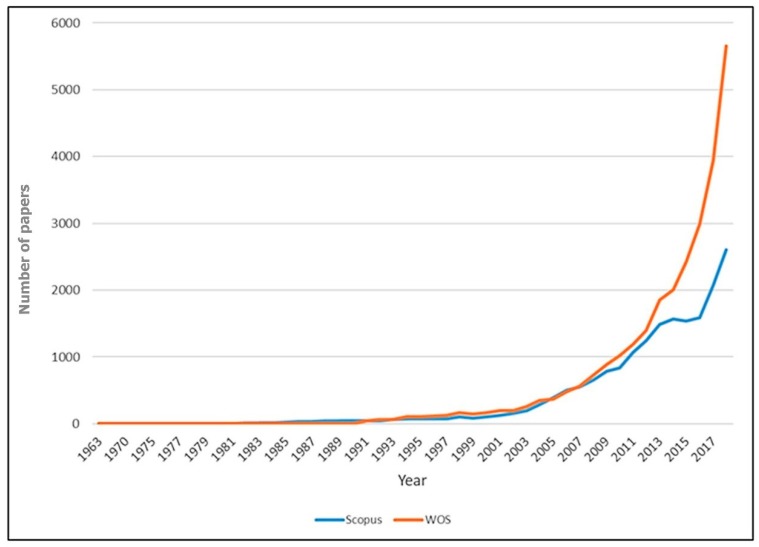
The number of papers by year in the database, 1977–2017.

Figure 2 is the visualization of this exponential growth of AI research in medicine. Although the number of papers in Web of Science is more than that in Scopus, both showed a similar trend over the study period. The number of publications has increased exponentially since 1998, and most of the papers (65.0% Scopus paper and 97.7% WoS paper) were published in 2008–2018. The first paper related to “AI in health and medicine” of WOS was found in 1977, whereas that of Scopus was in 1963.

**Figure 3:**
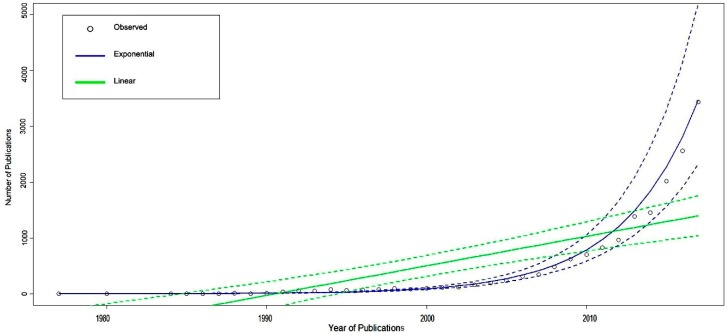
The number of papers since the year 1977 (estimated and observed). The dotted lines (corresponding in term of color to the two solid lines) represent the 95% confidence interval.

We attempted to estimate the number of publications related to AI in healthcare and medicine on a global level by employing an exponential model in which the dependent variable was the annual number of articles and the independent variable was the year published. The coefficients of determination (*r*^2^) of such model was 0.935. Figure 3 visualizes how the exponential model fit with observed data compared to a linear model. The dotted lines corresponding to each solid line in terms of color (dotted blue with solid blue, dotted green with solid green) represent the 95% confidence interval.

There is an inflection point in the amount of AI research that happens around 2002–2003 when the quantity of AI research in health and in medicine surges upward dramatically. This observation can be explained by the exponential growth of computing power and data storage capacity, which also went through an inflection point during the same period [2,24,25]. The revolution in computing power and digitalization has not only changed the quantity of research but also enabled a robot called Adam to identify the function of a yeast gene on 12 June 2007, a noteworthy point in the history of AI, as it effectively ended the human monopoly of scientific discovery [26].

Five thousand and fifty-seven journals published 27,451 research articles. Two-thousand two hundred and fifty-seven (8.2%) journals published one paper, 848 (3.09%) journals published two, 490 (1.78%) journals published three, and 1462 (86.9%) journals published four or more. PLOS One (*n* = 478; 1.74%) and Expert Systems with Applications (*n* = 281; 1.02%) are two journals that published the most papers, followed by Journal of Biomedical Informatics (*n* = 269; 1.00%), Medical Physics (*n* = 258; 0.94%), and Journal of Robotic Surgery (*n* = 255; 0.93%). “Expert systems with applications” (281 papers) was the most prolific journal, publishing in the categories “Computer Science” and “Engineering”. Among all topics, AI in health and medicine was the one which attracted the greatest concern, mainly from the medical community, which could be clearly seen from the diversity of the subject categories, such as surgery, medical chemistry, and oncology. (see Table S3 Supplementary).

### 3.2. Contribution by Author

As Table 1 shows, 40% (*n* = 10,992) of the papers were the fruits of collaboration of four or six authors; the number of papers with two or three authors was 8085 (29.5%) and only 5.15% (*n* = 1413) of items were written by one author. Given that publications in this field were mostly the results of coauthorship, one implication stands out here: conducting research on medical AI often requires extensive teamwork. This observation also highlights the multidisciplinary links among the authors and the interdisciplinary nature of the field. However, it is also noteworthy that the number of papers with more than 10 authors accounts for only 6.25% (*n* = 1724). This suggests it might not be effective for too many authors to collaborate on international publications; the data imply four to six authors as the optimal number of team members.

**Figure 4:**
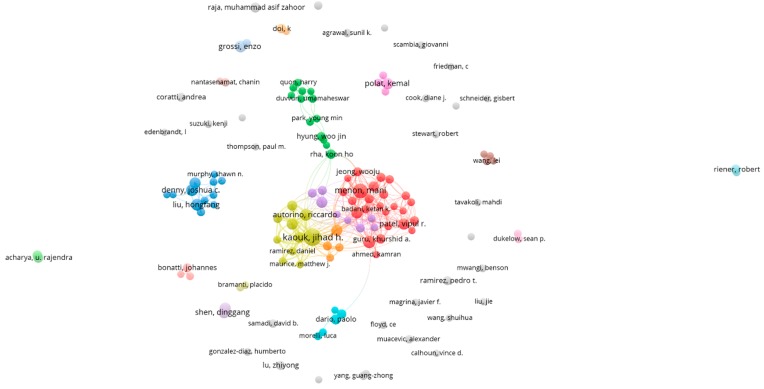
The global network of coauthors.

Applying the cut-off of 15 papers for one author, we visualized the global cooperation of authors. Among 135 authors in Figure 4, most of the prolific authors had strong collaborations with others and appeared at the center of many constellations. Such authors are Mani Menon (red cluster), Kaouk Jihad H, Autorino Ricardo (yellow cluster), and Inderbir S. Gill (purple cluster). Meanwhile, stand-alone authors had fewer papers. The thickness of lines is an indication of the strength of the relationship between authors relative to others. The strength of these relationships was determined by the frequency with which they appeared together in published articles. Their inclusion into specific thematic groups was based on their clustering with a certain constellation of terms. The position of an author within this constellation represents how interrelated and frequent their co-occurrence was with other authors. This pattern has been confirmed by other scientometric studies as socially important and productive researchers tend to drive the productivity of their coauthors [18,27].

### 3.3. Global Collaboration

Table 2 illustrates the productivity ranking of the top 20 countries in this dataset. In this list, the top five countries were from North America (the United States and Canada), Europe (Italy, and Germany), and China. The United States ranked at the top of all indices: total papers (10,623 papers, 30.8%), total citations (232,669 citations), and number of downloads (25,384). Countries in Europe had the strong co-operation in AIM, confirmed by the high number of institutes (>7.7), countries in collaboration (>2.0), and collaborative papers (>50%). Meanwhile, in Asia, China was ranked second with 2671 papers (7.6%) and 15,995 downloads. It is remarkable that Israel and Singapore had the highest level of international collaboration (nearly 70%), while the figure in other Asian countries was only one-third of this.

**Figure 5:**
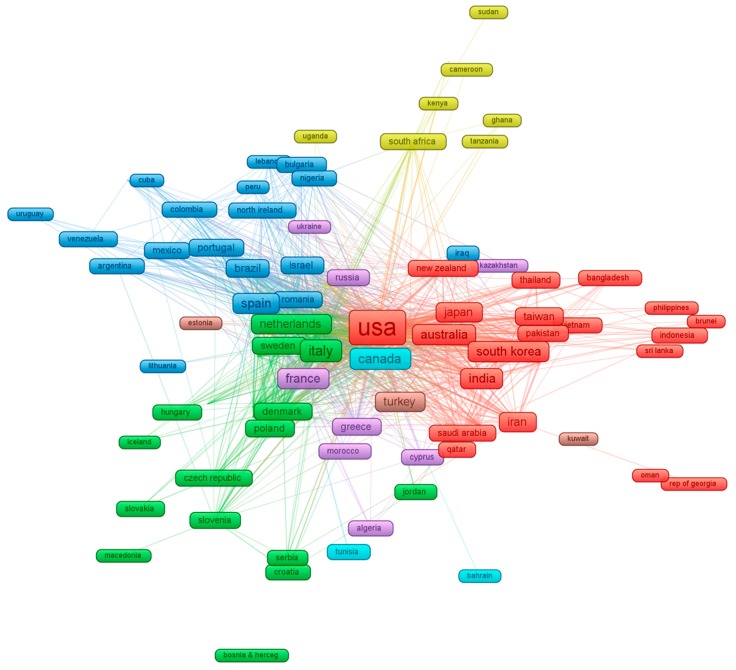
The global network of the 93 countries (at least five papers). Note: Figure 5 visually represents the proportional contributions of each country, including their collaborations with one another. The size of each country node represents this proportional contribution of articles to the data set. Attribution of papers to a country was based on the institutional affiliation of the lead author. The length of the lines was automatically generated and was based on the strength of the collaboration between two countries.

The network of 93 countries with the minimum of five papers is visually mapped and presented in Figure 5. This visualization also demonstrates the strength of collaborative partnerships between countries. Regarding AI technology, the United States, China, England, and Canada are leading the way. The red cluster showed the close collaboration between The United States and China, Australia, and other Asian countries, such as Japan, Taiwan, and South Korea. Europe sought to narrow the gap in the AI world leader race [28]. The largest cluster in Europe was formed among Germany, Italy, the Netherlands, and Denmark (green cluster). Clusters of collaboration were also seen among France, Greece, and Morocco (Purple cluster) or, beyond the European border, Spain was the leader in the cooperation with South America countries (Brazil, Mexico, and Argentina).

Table S1 (Supplementary) presents the most active institutions in AI technology publications, which also shows the leading positions of those from the United States, China, England and Canada.

### 3.4. Keyword and Text Analysis

**Figure 6:**
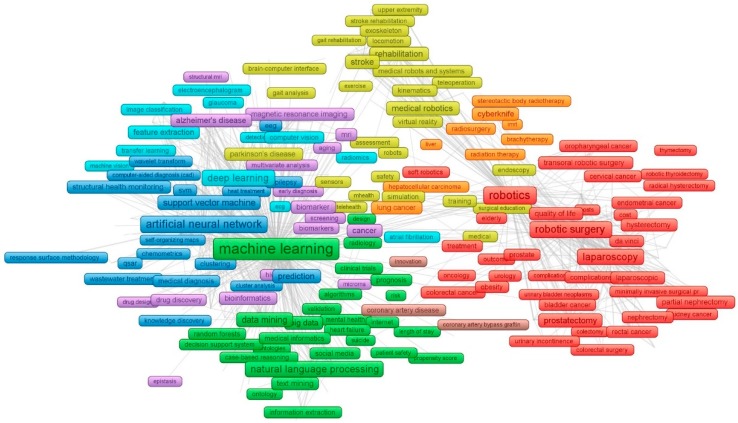
The co-occurrence of authors’ keywords. Figure 6 presents the prevalence of 568 keywords appearing in our search results using the Web of Science. The thickness of lines is an indication of the strength of the relationship between keywords relative to the others. The strength of these relationships was determined by the frequency with which they appeared together in published articles. Their inclusion into specific thematic groups was based on their clustering with a certain constellation of terms. The position of a keyword within this constellation represents how interrelated and frequent its co-occurrence was with other terms.

As can been seen from Figure 6, the author’s keywords can be divided into two groups: (1) types of AI, and (2) AI applications in health and medicine.

1. Types of AI: among all the keywords applied for searching, three kinds of AI being outstanding compared with others were “machine learning”, “robotics”, and “deep learning”
2. AI applications in health and medicine: For example, “machine learning”, “artificial intelligence”, “support vector machines” were used to support the diagnose and/or treatment of “Parkinson’s disease”, “Alzheimer’s disease” or used in “neuroimaging”. “Robotics” was utilized mainly for assisting laparoscopy of “oropharyngeal cancer” or “cervical cancer” or “surgery”. “Natural language processing” was applied for collecting “health records” information contributing to “big data” system.

It is quite remarkable that the keyword “ethics” is nowhere to be seen in the figure, suggesting that there is a lack of attention toward AI ethics in health and medicine. Additionally, in our dataset, when searching “ethics” on both keyword field and abstracts there are only 204 papers (0.7%) related to ethics. The first paper was published in 1994 “Ethical considerations in the management of individuals with severe neuromuscular disorders”. The application of AI has brought many benefits to the healthcare system and improve medicine. However, the use of AI technology unethically may be dangerous to patients and physicians. Thus, we need an ethical standard to apply to all the actors not only in healthcare services, but also in health-related fields [29].

Table 3 provides the number of publications of most common AI tools and types of clinical application using AI (diagnosis/prediction or treatment) for each of the top 25 diseases in terms of the burden of disease measured in DALYs [30]. Robotics is transforming health care with its diverse applications, such as early detection, training future doctors, or treatment. The highest number of papers were about robotic surgery. Machine learning was the second most popular AI type in which one would use for large and complex data analysis fields, such as genetics [31]. AI clinicians are higher than medical clinicians [32], however, in our dataset, the number of papers using AI for prediction of disease or its consequences was higher than that of treatment. The burdens of disease shift from infectious diseases to noncommunicational diseases (NCDs) [33], thus the research on AI application for heart disease, stroke, or respiratory disease were higher than for other diseases. The increasing burden due to cancer has attracted scientific concern, with the highest total number of papers in AI tools (Robotic, Machine leaning, or Artificial neural network) and in treatment and diagnosis. Robotics received the highest concern due to its broad application in medicine and health care, from serving as a nurse to supporting in surgery [34].

## 4. Discussion

To the best of our understanding, although bibliometrics has been used to explore the trends in other research areas [16,17,18,19,20,21], this study can be considered the first intensive global mapping and analysis of scientific research on AI in health and medicine.

The growth of scientific literature in the field of AI has increased rapidly, particularly in the past 10 years, thanks to the exponential growth of computing power and data storage capacity [2,25,26]. This growth is attributed to the prolific output of research at leading institutions located in the United States, Europe, and China. These three players are also the biggest contributors to overall AI research worldwide [2]. At the national level, this study points out that among the top 10 researchers, the number of citations per paper of Asian researchers is significantly lower than that of their North American peers. This can be explained by the late-coming of China to the field. In-depth research should look into factors driving the differences in research output and citation impacts between the two regions.

Our research was the first study showing the number of papers with AI applications in healthcare and medicine with the global burden of disease measured by DALYs. The number of papers related to disease with high rank in burden of disease shows there is shifting focus from infectious diseases to NCDs. The volume of publications mentioned about AI application in cancer was the highest, although the rank of cancer was 12th in the list (Table 3). The special concern that the scientific community spent for the second leading cause of death disease can be explained by (1) the uncertainty in early diagnosis and treatment outcomes; (2) the severity of late treatment, (3) the variety in types of cancers. Using AI in treatment will increase the level of accuracy, which may cause evolution of cancer treatment.

The absence of the word “ethics” in the research subjects, the keywords, and the text mining of the abstracts (Figure 5 and Figure S2 Supplementary) suggests the promotion of evidence-informed policy making, health system strengthening, and a renewed focus to ensure that AI should be developed and used in the transparent and accountable way, which is consistent with public interest. This suggests a need for research on AI-related policy. The effect of AI might be reduced where data are not available, difficult to collect, or transfer digitally.

Another implication is the factors contributing to research output in medical AI. Empirical data suggest influential authors, as measured by the total number of citations and the number of citations per paper, are often those who either lead a field and stay productive throughout his or her career, or invents a method applicable in a variety of research areas. This pattern has been observed in other studies, such that senior and productive authors will drive the productivity of their collaborators [28].

In terms of policy implications, this study puts forth three suggestions. First, recent approaches that are rising in popularity include the use of AI to collect health data and information, in support of treating cancer (Table 3). This means applications of AI in medicine will be increasingly useful in aiding diagnosis and clinical treatment. Second, developing countries should look to investment in research in medical AI. particularly China and India, which are emerging as top players in the field. Third, rapidly development of AI might create new challenges to established frameworks. Fourth, future AI techniques and development trends will focus on machine learning based on data obtained from the latest diagnostic modalities, including multi-omics (e.g., genomics, metabolomics) [35] and state-of-the-art imaging methods to predict treatment responses [36,37], especially in areas where there is a lack of objective diagnostic methods, e.g., psychiatric disorders [38]. Finally, to accelerate application and expansion of AI in health and medicine, it is critical to develop global and national protocols and regulations to frequently review and justify the validity of AIM products in clinical and practical environments.

An important challenge in applying AI for health and medicine is the lack of large clinical datasets for training AI models. This is especially true for datasets with labels, which require doctors/medical expert annotation and therefore are very costly and time-consuming to collect [39,40,41]. In general, AI techniques that are applicable to situations with limited amounts of labeled training data are of great interest for many applications [42], in addition to health and medicine. This is an important fundamental problem with active AI research and several promising AI directions: First, more sophisticated data augmentation techniques have recently been proposed to enrich the training datasets to better characterize data distribution. These techniques include feature space data augmentation [42,43] and data synthesis using complex deep neural networks models, notably generative adversarial networks (GAN) [44,45,46,47]. Second, there are substantial interests to improve semisupervised learning: learning methods that typically use a small amount of labeled data and unlabeled data [48]. Third, self-supervised learning [49,50] has attracted a lot of attention. Self-supervised learning methods automatically identify and extract supervisory signals without using any labeled data. Self-supervised learned models represent strong baselines that can be used in different applications with limited labeled data, including many medical and health applications.

Regarding the weaknesses and limitations of this study, the restriction on searchable peer-reviewed research publication and the exclusion of other documents may impact the thoroughness of the results and analysis. In addition, as only English articles and reviews were included in this study, the non-English papers were not counted. That made the number of Western countries’ publications, especially English-speaking ones, more than that of Asia or Africa. Nonetheless, a bibliometric analysis of a large volume of publications and a summary of keywords is a helpful proxy for the overall content of these papers. Further studies may benefit from investigating how different AI techniques are being used to resolve specific medical tasks or exploring the impacts of methodological versus more application-oriented AI studies.

Given the limitations and current scope of the study, both academics and practitioners are aware that the AI developments—despite the field’s vibrant growth—will further evolve in line with rising uncertainties and complexities in the coming years. It is hard to tell if one specific technique will prevail, but the surging trend is inexorable, to the extent that entrepreneurial attempts and policymaking changes will have to adapt. This study provides a contextual outline which may become useful for creating a more enabling environment, in which both AI developers and health researchers will work. It is anticipated that more in-depth reviews and theoretical surveys will be conducted, and early “maps” will more likely help at this early stage of development, let alone the fact that the recent debates on ethical issues of AI in the industries in general, and in the medical realm, in particular, will be less fruitful without a more updated understanding of macro views.

In conclusion, AI has been applied for a wide range of purposes, especially in in the field of healthcare. With the rapid development of technology, AI has the opportunity to help raise important health problems to light but might be restricted by the unavailability of health data, and/or by the inability of AI to have some human characteristics, such as compassion. The use of AI raises some ethical and social issues, which might be overcome via data policy. A key challenge for governments is that AI development should be conducted in a way that is easy to approach and aligned with the public interest.

## Figures and Tables

**Table 1:** Characteristics of the selected articles.

Characteristic	Category	Number	Percent
Total number of papers		27,451	100
Year of publication	2018–2014	16,913	61.62
	2013–2009	6303	22.97
	2008–2004	2471	9.00
	2003–1999	968	3.53
	1998–1994	599	2.18
	1993–1989	174	0.64
	<1989	23	0.06
Number of authors	1	1413	5.15
	2–3	8085	29.45
	4–6	10,992	40.05
	7–10	5237	19.07
	>10	1724	6.28
Number of subject categories	1	14,756	53.75
	2	7225	26.32
	3	3597	13.10
	4	1244	4.53
	>5	629	2.30
Number of countries in authorship	1	18,532	67.51
	2	4954	18.05
	3	1126	4.1
	4	295	1.07
	5	109	0.4
	6	42	0.15
	7	32	0.12
	8	20	0.07
	9	6	0.02
	>10	18	0.06

**Table 2:** The most prolific countries in AI in Health/Medicine research and their collaborations.

No.	Country	Total Papers	% Papers	Total Citations	Cite Rate	Total Downloads	Total Co-Authors	Total Institutes	Total Country	% Papers with International Collaboration
1	United States	10,623	30.8	232,669	3.4	25,384	5.8	6.8	1.5	33.6%
2	China	2617	7.6	27,997	2.9	15,995	5.9	7.3	1.7	44.8%
3	Italy	1834	5.3	29,485	2.8	3343	7.4	8.4	2.1	55.2%
4	Germany	1553	4.5	31,219	3.3	3415	7.4	8.9	2.1	53.1%
5	Canada	1312	3.8	22,608	2.9	3343	6.2	8.0	1.8	48.5%
6	France	1308	3.8	22,687	3.1	2623	7.4	9.0	2.2	63.7%
7	India	1264	3.7	12,871	2.1	3350	4.5	4.9	1.6	31.6%
8	Spain	1029	3.0	14,653	2.6	2852	6.5	7.7	2.0	49.3%
9	Australia	910	2.6	17,413	3.5	3337	5.8	7.5	2.0	50.5%
10	Japan	841	2.4	11,054	2.2	2107	6.7	7.5	1.7	35.7%
11	Turkey	787	2.3	9058	1.8	1121	4.1	4.4	1.4	20.3%
12	Iran	713	2.1	7438	2.2	1599	4.1	4.6	1.4	30.2%
13	Netherlands	640	1.9	14,811	4.4	1948	8.0	10.1	2.4	59.2%
14	Switzerland	554	1.6	10,197	3.6	1804	7.6	9.7	2.3	63.5%
15	Taiwan	543	1.6	6213	1.8	1084	5.1	6.6	1.5	28.5%
16	Brazil	489	1.4	6097	2.6	1380	6.6	8.1	1.9	44.6%
17	Israel	384	1.1	6393	3.4	950	7.8	9.8	2.4	69.8%
18	Sweden	382	1.1	6220	3.0	700	7.7	9.7	2.4	61.3%
19	Belgium	365	1.1	7269	3.5	696	8.8	10.7	2.8	66.6%
20	Singapore	349	1.0	5378	3.2	1904	6.0	7.1	2.2	66.2%

**Table 3:** Number of papers related to AI techniques, clinical application of AI, and top burden of diseases.

Title	Robotic	Machine Learning	Artificial Neural Network	Artificial Intelligence	Natural Language Process	Deep Learning	Fuzzy Expert System	Evolutionary Computation	Clinical Application	Treatment	Prediction	Diagnosis	Total	Rank Burden of Diseases (DALY)
Cancer	1774	767	366	144	103	0	5	6	28	209	1008	82	4492	12
Heart Diseases	263	357	166	75	33	59	11	3	11	19	331	33	1361	1
Vision	543	264	81	101	57	1	4	5	19	27	189	21	1312	22
Stroke	543	99	45	16	9	0	0	2	13	32	119	5	883	3
Alzheimer	17	314	70	18	8	0	0	1	5	2	210	48	693	24
Depression	71	232	29	33	24	0	1	0	7	39	178	6	620	20
Kidney	293	64	38	14	9	0	2	0	1	17	125	9	572	19
Diabetes	38	159	86	34	20	0	4	3	1	6	176	21	548	8
Respiratory	119	104	64	20	16	2	0	0	1	9	136	6	477	4
Substance use	75	101	57	17	35	7	0	0	2	20	120	4	438	16
HIV	24	114	35	28	21	6	0	2	6	10	113	4	363	13
Injuries	162	32	9	9	5	0	1	0	5	7	30	1	261	5
Asthma	12	60	29	6	12	1	2	0	0	0	62	4	188	25
Tuberculosis	8	42	17	12	2	2	0	0	1	2	42	15	143	14
Congenital	64	24	14	5	1	3	0	0	3	6	19	3	142	9
Cirrhosis	11	23	24	6	2	0	0	0	2	4	38	2	112	11
Malaria	4	38	9	8	1	0	1	0	1	0	42	2	106	15
Suicide	0	43	1	3	10	0	0	0	1	1	34	1	94	16
COPD	5	32	7	3	3	0	1	0	0	0	29	1	81	6
Neonatal	7	22	8	5	4	3	1	0	0	0	22	1	73	2
Hearing	7	11	9	6	0	0	0	1	0	1	14	3	52	21
Back pain	18	5	9	2	1	0	0	0	2	2	11	0	50	10
Headache disorders	0	0	0	0	0	5	0	0	0	0	0	0	5	23
Diarrhea	1	0	0	0	0	0	0	0	0	0	0	0	1	7
Headache disorders	0	0	0	0	0	1	0	0	0	0	0	0	1	18
Total	4059	2907	1173	565	376	90	33	23	109	413	3048	272	13,068	

## Supplementary Materials

The following are available online at https://www.mdpi.com/2077-0383/8/3/360/s1, Table S1: Search results (WOS), Table S2: Search results (Scopus), Table S3: Journal subject category and the Journal included those subject categories, Figure S1: Institutions network, Figure S2: Text (titles and abstracts) mining.
