# Supplementary material for: Global Evolution of Research in Artificial Intelligence in Health and Medicine: A Bibliometric Study
Source: J Clin Med. 2019 Mar 14;8(3):360. doi: 10.3390/jcm8030360 (PMC6463262; doi:10.3390/jcm8030360)
Supplement: Supplementary file 1 [file jcm-08-00360-s001.pdf]

**Supplementary**

**Table S1.** Search results (WOS)

| Number | Search query | Result |
|---|---|---|
| #1 | TS=("Artificial intelligence" OR "Machine intelligence" OR "artificial neural network*" OR "Machine learning" OR "Deep learn*" OR "Natural language process*"OR "Robotic*" OR "thinking computer system" OR "fuzzy expert system*" OR "evolutionary computation" OR "hybrid intelligent system*") | 332,892 |
| #2 | TS=(disease* OR illness OR health-related OR medic* OR "medical diagnosis" OR treatment OR health* OR wellness OR well-being) | 10,609,911 |
| #3 | #2 AND #1 | 44,519 |
| #4 | #2 AND #1<br>Refined by: [excluding] PUBLICATION YEARS: ( 2019 ) | 43,768 |
| #5 | #2 AND #1<br>Refined by: [excluding] PUBLICATION YEARS: ( 2019 ) AND [excluding] DOCUMENT TYPES: ( PROCEEDINGS PAPER OR BOOK CHAPTER OR MEETING ABSTRACT OR EDITORIAL MATERIAL OR LETTER OR NEWS ITEM OR BOOK OR BOOK REVIEW OR CORRECTION OR DATA PAPER OR EARLY ACCESS OR NOTE OR REPRINT OR BIOGRAPHICAL ITEM OR RETRACTED PUBLICATION OR DATABASE REVIEW ) | 28,344 |
| #6 | #2 AND #1<br>Refined by: [excluding] PUBLICATION YEARS: ( 2019 ) AND [excluding] DOCUMENT TYPES: ( PROCEEDINGS PAPER OR BOOK CHAPTER OR MEETING ABSTRACT OR EDITORIAL MATERIAL OR LETTER OR NEWS ITEM OR BOOK OR BOOK REVIEW OR CORRECTION OR DATA PAPER OR | 27,617 |

| | | |
|---|---|---|
| | EARLY ACCESS OR NOTE OR REPRINT OR BIOGRAPHICAL ITEM OR RETRACTED PUBLICATION OR DATABASE REVIEW ) AND [excluding] LANGUAGES: ( GERMAN OR SPANISH OR FRENCH OR CHINESE OR PORTUGUESE OR TURKISH OR RUSSIAN OR KOREAN OR ITALIAN OR POLISH OR CZECH OR HUNGARIAN OR CROATIAN OR JAPANESE OR SLOVENIAN OR CATALAN OR ROMANIAN OR UKRAINIAN OR MALAY OR SLOVAK ) | |
| #7 | AU=Anonymous | 1,406,134 |
| #8 | #6 NOT #7 | 27,607 |
| #9 | #8 Refined by: [excluding] Undefined data | 27,451 |

**Table S2. Search results (Scopus)**

| | **Search query** | **Result** |
|---|---|---|
| 1 | TITLE-ABS-KEY ( "Artificial intelligence" OR "Machine intelligence" OR "artificial neutral network?" OR "Machine learning" OR "Deep learn?" OR "Natural language process?" OR "Robotic?" OR "thinking computer system" OR "fuzzy expert system?" OR "evolutionary computation" OR "hybrid intelligent system?" ) | 574,772 |
| 2 | TITLE-ABS-KEY ( disease? OR illness OR health-related OR medic? OR "medical diagnosis" OR treatment OR health? OR wellness OR well-being ) | 11,022,297 |
| 3 | ( TITLE-ABS-KEY ( "Artificial intelligence" OR "Machine intelligence" OR "artificial neutral network?" OR "Machine learning" OR "Deep learn?" OR "Natural language process?" OR "Robotic?" OR "thinking computer system" OR "fuzzy expert system?" OR "evolutionary computation" OR "hybrid intelligent system?" ) ) AND ( TITLE-ABS-KEY ( disease? OR illness OR | 36,266 |

| | | |
|---|---|---|
| | health-related OR medic? OR "medical diagnosis" OR treatment OR health? OR wellness OR well-being ) ) | |
| 4 | ( TITLE-ABS-KEY ( "Artificial intelligence" OR "Machine intelligence" OR "artificial neutral network?" OR "Machine learning" OR "Deep learn?" OR "Natural language process?" OR "Robotic?" OR "thinking computer system" OR "fuzzy expert system?" OR "evolutionary computation" OR "hybrid intelligent system?" ) ) AND ( TITLE-ABS-KEY ( disease? OR illness OR health-related OR medic? OR "medical diagnosis" OR treatment OR health? OR wellness OR well-being ) ) AND ( EXCLUDE ( PREFNAMEAUID , "Undefined#Undefined" ) ) AND ( EXCLUDE ( PUBYEAR , 2019 ) | 35,386 |
| 5 | ( TITLE-ABS-KEY ( "Artificial intelligence" OR "Machine intelligence" OR "artificial neutral network?" OR "Machine learning" OR "Deep learn?" OR "Natural language process?" OR "Robotic?" OR "thinking computer system" OR "fuzzy expert system?" OR "evolutionary computation" OR "hybrid intelligent system?" ) ) AND ( TITLE-ABS-KEY ( disease? OR illness OR health-related OR medic? OR "medical diagnosis" OR treatment OR health? OR wellness OR well-being ) ) AND ( EXCLUDE ( PUBYEAR , 2019 ) ) AND ( EXCLUDE ( PREFNAMEAUID , "Undefined#Undefined" ) ) | 35,386 |
| 6 | ( TITLE-ABS-KEY ( "Artificial intelligence" OR "Machine intelligence" OR "artificial neutral network?" OR "Machine learning" OR "Deep learn?" OR "Natural language process?" OR "Robotic?" OR "thinking computer system" OR "fuzzy expert system?" OR "evolutionary computation" OR "hybrid intelligent system?" ) ) AND ( TITLE-ABS-KEY ( disease? OR illness OR health-related OR medic? OR "medical diagnosis" OR treatment OR health? OR wellness OR well-being ) ) AND ( EXCLUDE ( PUBYEAR , 2019 ) ) AND ( EXCLUDE ( PREFNAMEAUID , "Undefined#Undefined" ) ) AND ( EXCLUDE ( SUBJAREA , "Undefined" ) ) | 35,341 |

| | | |
|---|---|---|
| 7 | ( TITLE-ABS-KEY ( "Artificial intelligence" OR "Machine intelligence" OR "artificial neutral network?" OR "Machine learning" OR "Deep learn?" OR "Natural language process?" OR "Robotic?" OR "thinking computer system" OR "fuzzy expert system?" OR "evolutionary computation" OR "hybrid intelligent system?" ) ) AND ( TITLE-ABS-KEY ( disease? OR illness OR health-related OR medic? OR "medical diagnosis" OR treatment OR health? OR wellness OR well-being ) ) AND ( EXCLUDE ( PUBYEAR , 2019 ) ) AND ( EXCLUDE ( PREFNAMEAUID , "Undefined#Undefined" ) ) AND ( EXCLUDE ( SUBJAREA , "Undefined" ) ) AND ( EXCLUDE ( DOCTYPE , "cp" ) OR EXCLUDE ( DOCTYPE , "ch" ) OR EXCLUDE ( DOCTYPE , "no" ) OR EXCLUDE ( DOCTYPE , "ed" ) OR EXCLUDE ( DOCTYPE , "cr" ) OR EXCLUDE ( DOCTYPE , "ip" ) OR EXCLUDE ( DOCTYPE , "le" ) OR EXCLUDE ( DOCTYPE , "sh" ) OR EXCLUDE ( DOCTYPE , "bk" ) OR EXCLUDE ( DOCTYPE , "er" ) OR EXCLUDE ( DOCTYPE , "rp" ) ) | 20,142 |
| 8 | ( TITLE-ABS-KEY ( "Artificial intelligence" OR "Machine intelligence" OR "artificial neutral network?" OR "Machine learning" OR "Deep learn?" OR "Natural language process?" OR "Robotic?" OR "thinking computer system" OR "fuzzy expert system?" OR "evolutionary computation" OR "hybrid intelligent system?" ) ) AND ( TITLE-ABS-KEY ( disease? OR illness OR health-related OR medic? OR "medical diagnosis" OR treatment OR health? OR wellness OR well-being ) ) AND ( EXCLUDE ( PUBYEAR , 2019 ) ) AND ( EXCLUDE ( PREFNAMEAUID , "Undefined#Undefined" ) ) AND ( EXCLUDE ( SUBJAREA , "Undefined" ) ) AND ( EXCLUDE ( DOCTYPE , "cp" ) OR EXCLUDE ( DOCTYPE , "ch" ) OR EXCLUDE ( DOCTYPE , "no" ) OR EXCLUDE ( DOCTYPE , "ed" ) OR EXCLUDE ( DOCTYPE , "cr" ) OR EXCLUDE ( DOCTYPE , "ip" ) OR EXCLUDE ( DOCTYPE , "le" ) OR EXCLUDE ( DOCTYPE , "sh" ) OR EXCLUDE ( DOCTYPE , "bk" ) OR | 19,711 |

| | | |
|---|---|---|
| | EXCLUDE ( DOCTYPE , "er" ) OR EXCLUDE ( DOCTYPE , "rp" ) ) AND ( EXCLUDE ( SRCTYPE , "k" ) OR EXCLUDE ( SRCTYPE , "d" ) OR EXCLUDE ( SRCTYPE , "p" ) OR EXCLUDE ( SRCTYPE , "b" ) OR EXCLUDE ( SRCTYPE , "Undefined" ) ) | |
| 9 | ( TITLE-ABS-KEY ( "Artificial intelligence" OR "Machine intelligence" OR "artificial neutral network?" OR "Machine learning" OR "Deep learn?" OR "Natural language process?" OR "Robotic?" OR "thinking computer system" OR "fuzzy expert system?" OR "evolutionary computation" OR "hybrid intelligent system?" ) ) AND ( TITLE-ABS-KEY ( disease? OR illness OR health-related OR medic? OR "medical diagnosis" OR treatment OR health? OR wellness OR well-being ) ) AND ( EXCLUDE ( PUBYEAR , 2019 ) ) AND ( EXCLUDE ( PREFNAMEAUID , "Undefined#Undefined" ) ) AND ( EXCLUDE ( SUBJAREA , "Undefined" ) ) AND ( EXCLUDE ( DOCTYPE , "cp" ) OR EXCLUDE ( DOCTYPE , "ch" ) OR EXCLUDE ( DOCTYPE , "no" ) OR EXCLUDE ( DOCTYPE , "ed" ) OR EXCLUDE ( DOCTYPE , "cr" ) OR EXCLUDE ( DOCTYPE , "ip" ) OR EXCLUDE ( DOCTYPE , "le" ) OR EXCLUDE ( DOCTYPE , "sh" ) OR EXCLUDE ( DOCTYPE , "bk" ) OR EXCLUDE ( DOCTYPE , "er" ) OR EXCLUDE ( DOCTYPE , "rp" ) ) AND ( EXCLUDE ( SRCTYPE , "k" ) OR EXCLUDE ( SRCTYPE , "d" ) OR EXCLUDE ( SRCTYPE , "p" ) OR EXCLUDE ( SRCTYPE , "b" ) OR EXCLUDE ( SRCTYPE , "Undefined" ) ) AND ( EXCLUDE ( LANGUAGE , "Chinese" ) OR EXCLUDE ( LANGUAGE , "German" ) OR EXCLUDE ( LANGUAGE , "French" ) OR EXCLUDE ( LANGUAGE , "Spanish" ) OR EXCLUDE ( LANGUAGE , "Japanese" ) OR EXCLUDE ( LANGUAGE , "Russian" ) OR EXCLUDE ( LANGUAGE , "Italian" ) OR EXCLUDE ( LANGUAGE , "Korean" ) OR EXCLUDE ( LANGUAGE , "Portuguese" ) OR EXCLUDE ( LANGUAGE , "Turkish" ) OR EXCLUDE ( LANGUAGE , "Czech" ) OR | 18,688 |

| | | |
|---|---|---|
| | EXCLUDE ( LANGUAGE , "Polish" ) OR EXCLUDE ( LANGUAGE , "Dutch" ) OR EXCLUDE ( LANGUAGE , "Hungarian" ) OR EXCLUDE ( LANGUAGE , "Persian" ) OR EXCLUDE ( LANGUAGE , "Romanian" ) OR EXCLUDE ( LANGUAGE , "Danish" ) OR EXCLUDE ( LANGUAGE , "Serbian" ) OR EXCLUDE ( LANGUAGE , "Croatian" ) OR EXCLUDE ( LANGUAGE , "Slovak" ) OR EXCLUDE ( LANGUAGE , "Finnish" ) OR EXCLUDE ( LANGUAGE , "Greek" ) OR EXCLUDE ( LANGUAGE , "Norwegian" ) OR EXCLUDE ( LANGUAGE , "Slovenian" ) OR EXCLUDE ( LANGUAGE , "Arabic" ) OR EXCLUDE ( LANGUAGE , "Hebrew" ) OR EXCLUDE ( LANGUAGE , "Swedish" ) OR EXCLUDE ( LANGUAGE , "Undefined" ) ) | |

**Table S3.** Journal subject category and the Journal included those subject categories.

| Research area | Total papers | Journal name | Total papers | Journal name | Total papers |
|---|---|---|---|---|---|
| Engineering | 5,037 | Expert systems with applications | 281 | Computer methods and programs in biomedicine | 186 |
| | | Artificial intelligence in medicine | 202 | IEEE transactions on biomedical engineering | 131 |
| | | Computers in biology and medicine | 187 | IEEE access | 128 |
| Computer science | 4,902 | Expert systems with applications | 281 | Artificial intelligence in medicine | 202 |
| | | Journal of biomedical informatics | 270 | Computers in biology and medicine | 187 |
| | | Journal of the American medical informatics association | 253 | Computer methods and programs in biomedicine | 186 |
| Surgery | 2,646 | Journal of robotic surgery | 260 | Asian journal of surgery | 85 |
| | | International journal of medical robotics and computer assisted surgery | 213 | JSLS journal of the society of laparoendoscopic surgeons | 79 |
| | | Surgical endoscopy and other interventional techniques | 190 | Journal of laparoendoscopic advanced surgical techniques | 76 |
| Medical informatics | 1,981 | Journal of biomedical informatics | 270 | Computer methods and programs in biomedicine | 186 |
| | | Journal of the American medical informatics association | 253 | Journal of medical systems | 158 |

| Research area | Total papers | Journal name | Total papers | Journal name | Total papers |
|---|---|---|---|---|---|
| | | Artificial intelligence in medicine | 202 | International journal of medical informatics | 103 |
| Neurosciences neurology | 1,759 | Journal of neuroengineering and rehabilitation | 116 | Neuroimage clinical | 48 |
| | | Neuroimage | 106 | Journal of neuroscience methods | 43 |
| | | Frontiers in neuroscience | 54 | Frontiers in neurology | 41 |
| Urology nephrology | 1,649 | Journal of urology | 175 | Urology | 134 |
| | | Journal of endourology | 161 | European urology | 128 |
| | | Bju international | 147 | Current opinion in urology | 82 |
| Radiology nuclear medicine medical imaging | 1,648 | Medical physics | 258 | Physics in medicine and biology | 85 |
| | | Neuroimage | 106 | International journal of computer assisted radiology and surgery | 73 |
| | | IEEE transactions on medical imaging | 92 | Journal of digital imaging | 66 |
| Science technology other topics | 1,385 | Plos one | 486 | Jove journal of visualized experiments | 34 |
| | | Scientific reports | 228 | Acs applied materials interfaces | 31 |
| | | Proceedings of the national academy of sciences of the united states of america | 42 | Advanced materials | 23 |
| Oncology | 1,306 | Gynecologic oncology | 63 | Technology in cancer research treatment | 53 |
| | | Annals of surgical oncology | 61 | Urologic oncology seminars and original investigations | 53 |

| Research area | Total papers | Journal name | Total papers | Journal name | Total papers |
|---|---|---|---|---|---|
| | | International journal of radiation oncology biology physics | 56 | EJSO | 36 |
| Mathematical computational biology | 1,228 | Computers in biology and medicine | 187 | Bioinformatics | 85 |
| | | BMC bioinformatics | 161 | Journal of medical imaging and health informatics | 64 |
| | | IEEE journal of biomedical and health informatics | 88 | Medical biological engineering computing | 59 |
| Biochemistry molecular biology | 1,210 | BMC bioinformatics | 161 | Plos computational biology | 43 |
| | | Bioinformatics | 85 | Journal of proteome research | 30 |
| | | Journal of neuroscience methods | 43 | Journal of biomedical optics | 28 |
| Chemistry | 1,181 | Sensors | 183 | Chemometrics and intelligent laboratory systems | 30 |
| | | Applied sciences Basel | 44 | Analytical chemistry | 26 |
| | | Journal of chemical information and modeling | 36 | Combinatorial chemistry high throughput screening | 26 |
| Health care sciences services | 1,176 | Journal of the American medical informatics association | 253 | Methods of information in medicine | 99 |
| | | Journal of medical systems | 158 | Journal of medical internet research | 87 |
| | | International journal of medical informatics | 103 | Journal of healthcare engineering | 31 |

| Research area | Total papers | Journal name | Total papers | Journal name | Total papers |
| --- | --- | --- | --- | --- | --- |
| Environmental sciences ecology | 762 | International journal of environmental research and public health | 28 | Environmental science and pollution research | 26 |
| | | Science of the total environment | 28 | Water research | 26 |
| | | Environmental modelling software | 27 | Water science and technology | 26 |

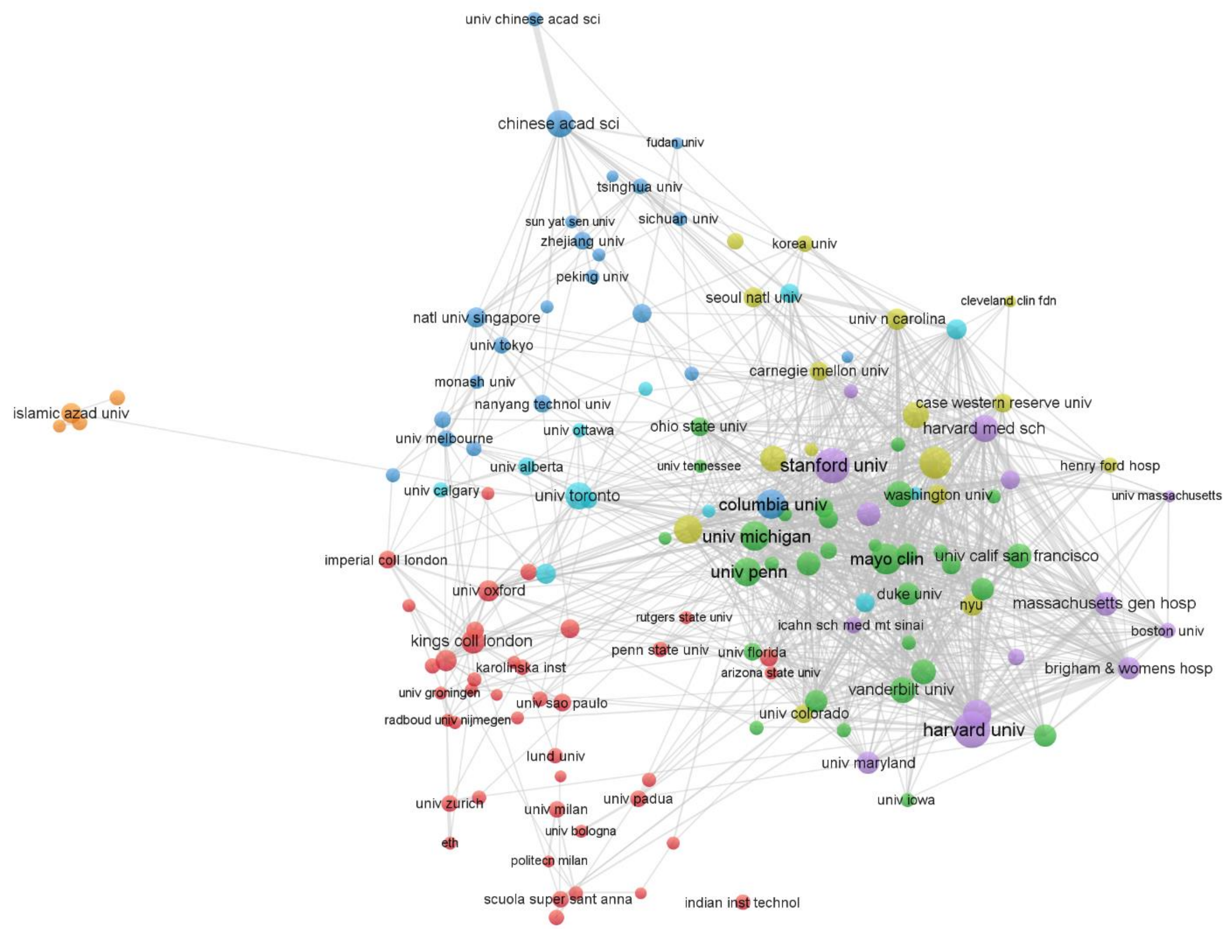


**Figure S1.** Institutions network. Figure S1 presents the network of institutions appearing in the Web of Science. In total of 16,930, we applied the cutoff point 60 papers, 142 institutions were in the figure. The thickness of lines is an indication of the strength of the relationship between institutions relative to the others. The strength of these relationships was determined by the frequency with which they appeared together in published articles. Their inclusion into specific thematic groups was based on their clustering with a certain constellation of terms. The position of an institution within this constellation represents how interrelated and frequent its co-occurrence was with others.

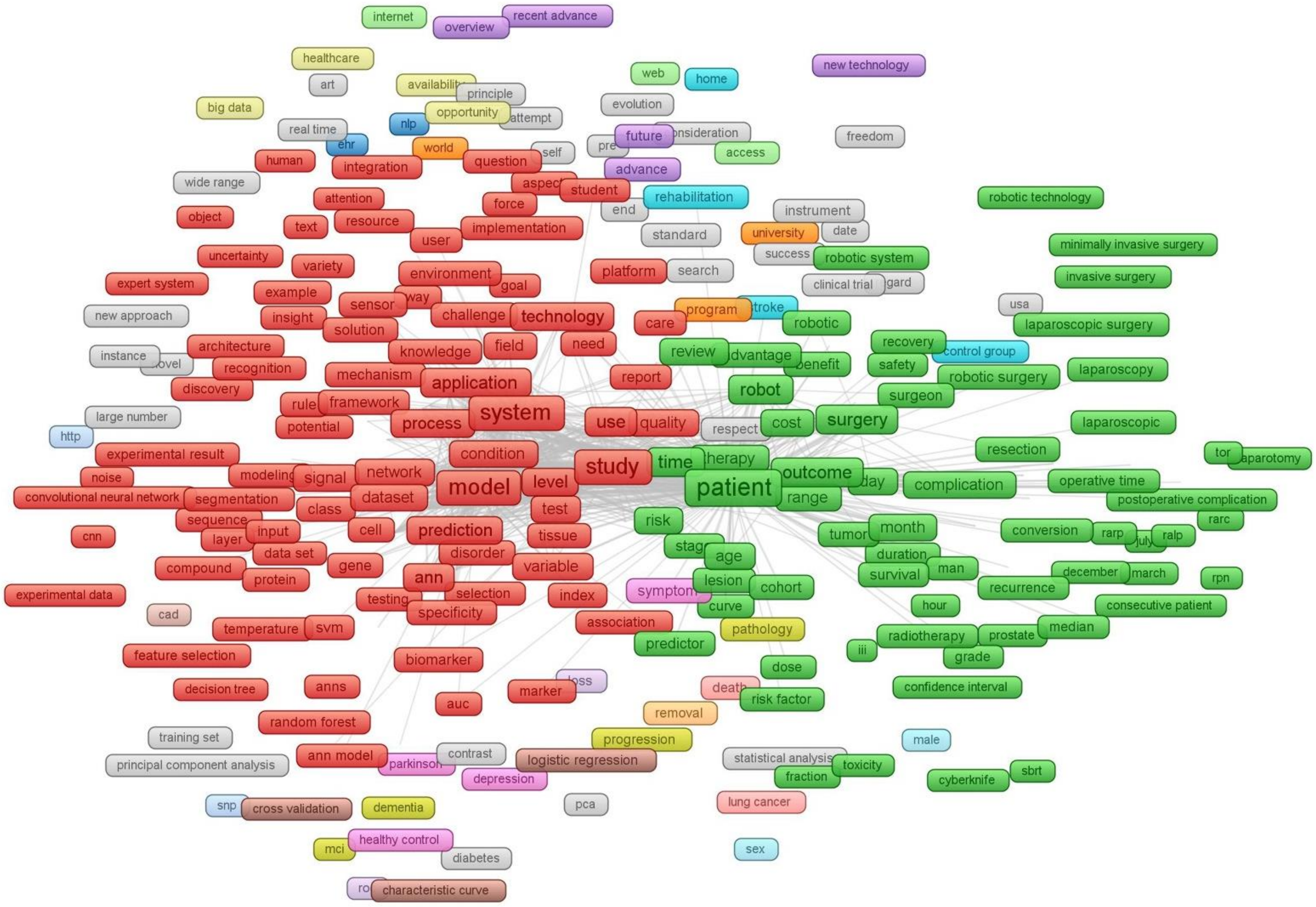


**Figure S2.** Text (titles and abstracts) mining. Figure S2 represents the prevalence of most frequent words appearing in title and abstract using Web of Science (550 terms with minimum 300 times of occurrences). The thickness of lines is an indication of the strength of the relationship between words relative to the others. The strength of these relationships was determined by the frequency with which they appeared together in published articles. Their inclusion into specific thematic groups was based on their clustering with a certain constellation of terms. The position of a key word within this constellation represents how interrelated and frequent its co-occurrence was with other term
